# The Occurrence of Zoonotic *Anaplasma phagocytophilum* Strains, in the Spleen and Liver of Wild Boars from North-West and Central Parts of Poland

**DOI:** 10.1007/s11686-021-00368-6

**Published:** 2021-03-26

**Authors:** Anna W. Myczka, T. Szewczyk, Z. Laskowski

**Affiliations:** Witold Stefański Institute of Parasitology, Polish Academy of Sciences, Twarda 51/55, 00-818 Warsaw, Poland

**Keywords:** *Anaplasma phagocytophilum*, *Sus scrofa*, Tick–borne disease, *groEL*, *ankA*

## Abstract

**Purpose:**

The *Anaplasma* genus includes a Gram-negative bacterium infecting the blood cells of wild and domestic mammals, causing tick-borne fever. Infection with pathogenic *Anaplasma phagocytophilum* strains may cause Human Granulocytic Anaplasmosis. Wild boars (*Sus scrofa*) may act as natural wild reservoir hosts for potentially zoonotic *A. phagocytophilum* strains; however, there is still little data to confirm this statement. The aim of this study was to verify whether wild boars can be classified as natural reservoirs of *Anaplasma* spp. and to compare the suitability of spleen and liver samples for such analysis.

**Methods:**

Liver and spleen samples were collected from 59 wild boars (2017–2019). The organs were tested for *Anaplasma phagocytophilum* using short (partial) fragments of three markers: 16S rRNA, *groEL*, *ankA*.

**Results:**

*Anaplasma* spp. DNA was detected in 12 out of 59 samples, with a prevalence of 20.34%. The presence of *A. phagocytophilum* was confirmed by sequencing of the partial 16S rRNA gene. Positive individuals were tested for the characteristic markers: *groEL* and *ankA*. The analysis of the nucleotide sequences of 16S rRNA, *groEL* and *ankA*, indicated that the strains of *A. phagocytophilum* detected in these studies are potentially zoonotic for humans.

**Conclusion:**

Wild boars from Poland can be classified as a natural reservoir of the zoonotic strain of *Anaplasma phagocytophilum*. Both the spleen and the liver tissues were found to be suitable materials for the detection of *A. phagocytophilum*.

## Introduction

The causative agent of Human Granulocytic Anaplasmosis (HGA), tick-borne fever (TBF) and granulocytic anaplasmosis in wild, domestic and farm animals is the parasitic bacterium *Anaplasma phagocytophilum* [1, 2]. *Anaplasma* spp. are gram-negative bacteria with a specific cell wall structure. *Anaplasma phagocytophilum* is an obligatory intracellular parasite that lives in neutrophils [3] and can be transmitted by ticks: *Ixodes ricinus, I. persulcatus, I. scapularis* and *Dermacentor reticulatus* [4–6]. Recent reports by Werszko et al. (2019) [7] show that the blood-sucking flies from Tabanidae family can act as carriers of *A. phagocytophilum*; however, more research is needed to confirm whether they can act as transmission vectors. Ticks and other hematophagous ectoparasites can easily transfer such bacterial intracellular parasites between natural animal reservoirs and humans [8, 9]. The considerable climate changes observed over the past 20 years have resulted in the spread of arthropods, such as ticks and flies, carrying pathogenic bacteria into new regions. Such an increase in annual temperatures has also led to a significant increase in the number of ectoparasites already present in the area, which significantly influences the spread of intracellular bacterial parasites, including *Anaplasma* spp., in the natural environment [10]. Many wild animals, such as roe deer (*Capreolus capreolus*), red deer (*Cervus elaphus*), wild boar (*Sus scrofa*), red fox (*Vulpes vulpes*), raccoon dog (*Nyctereutes procyonoides*) and the European badger (*Meles meles*) are infected with *A. phagocytophilum* [11, 12]. Wild boars are likely to be natural wild reservoir hosts for potentially zoonotic *A. phagocytophilum* strains; however, there is still little data to confirm this statement [11, 13].

The aim of this study was to verify whether the wild boar populations in the north-east and central parts of Poland can be natural reservoirs of *A. phagocytophilum*, a bacterium potentially pathogenic to humans. It also determines which of the internal organs collected from wild boar, i.e., spleen or liver, is more suitable for the detection of *Anaplasma phagocytophilum*.

## Materials and Methods

All materials were collected during the 2017/2018 hunting season in the Pisz Forest (Warmian-Masurian Voivodeship) and in the 2018/2019 hunting season in the Bolimów Forest (Łódź Voivodeship). In total, spleen and liver samples were collected from 59 adult wild boars.

DNA from both organs was isolated using a commercial DNA Mini Kit (Syngen). *Anaplasma* spp. was then detected using semi-nested PCR to amplify the partial 16S rRNA gene with primers specific to *Anaplasma* genus according to Szewczyk et al. (2019) [12]. Positive samples for *A. phagocytophilum* were additionally tested for the presence of the partial *groEL* and *ankA* genes with nested PCR according to Alberti el al. (2005), Massung et al. (2007) and Rymaszewska (2014), respectively (Table 1) [14–16]. DNA amplification was performed using the DNA Engine T100 Thermal Cycler (BioRad, USA). The PCR products were visualized on a 1.2% agarose gel (Promega, USA) stained with SimplySafe (EURx, Poland). Visualization was performed using ChemiDoc, MP Lab software (Imagine, BioRad, USA). The obtained PCR products were purified with the QIAquick Purification Kit (Qiagen, Germany). The purified products were sequenced directly using ABI BigDye™ chemistry (Applied Biosystems, USA) on an ABI Prism 373xl or an ABI Prism 3100™ automated sequencer. The obtained sequences were submitted to the GenBank.

**Table 1:** Primer used to amplify DNA markers of *A. phagocytophilum* in this study

Gene	Primers	References
*16r RNA*	A 500 F 5′CGTTGTTCGGAATTATTGGGCGTA-3′ A 520 F 5′-GGGCATGTAGGCGGTTCGGT-3′ A 900 R 5′-CCATGCAGCACCTGTGCGAG-3′	Szewczyk et al. 2019 [19]
*ankA*	ANK-F1 5′-GAAGAAATTACAACTCCTGAAG-3′ ANK-R1 5′-CAGCCAGATGCAGTAACGTG-3′ ANK-F2 5′-TTGACCGCTGAAGCACTAAC-3′ ANK-R2 5′-ACCATTTGCTTCTTGAGGAG-3′	Massung et al. 2007 and Rymaszewska 2014 [15, 16]
*groEL*	EphplgroEL569 F 5′-ATGGTATGCAGTTTGATCGC-3′ EphplgroEL1193 R 5′-TCTACTCTGTCTTTGCGTTC-3′ EphgroEL1142 R 5′-TTGAGTACAGCAACACCACCGGAA-3′	Alberti el al. 2005 [14]

## Results and discussion

Of the 59 wild boar from which spleen and liver samples were taken, DNA of *Anaplasma* spp. were detected in 12 individuals, i.e., a prevalence of 20.34%. *Anaplasma* spp. genetic material was detected in seven individuals in the spleen samples, and in six individuals in the liver samples (Table 2). All positive samples were obtained from boars in the Pisz Forest; no positive samples were found in the Bolimów Forest. Four positive samples were selected for sequencing, and the results indicated the presence of *A. phagocytophilum*.

**Table 2:** The presence of *Anaplasma* spp. molecular markers in wild boars

Biological material gene		SPLEEN	LIVER	SPLEEN and LIVER	Total
16S rRNA	7/59		6/59	1/59	12/59
*ankA*	2/59		2/59	0/59	4/59
*groEL*	2/59		2/59	1/59	3/59
*ankA* and *groEL*	2/59		1/59	0/59	3/59

A number of studies in various countries have been performed on wild boars with the aim of identifying natural reservoirs of zoonotic strains of *A. phagocytophilum* [2, 17, 18]. Most of these tests are based on analyses of blood and spleen samples [1, 2, 17, 19, 20] and, very rarely, liver samples [18, 21]. Although some individual studies have used both the spleen and liver [21, 22] none indicate which is more suitable for this type of analysis. Our findings clearly show that, in wild boars, both these tissues are suitable (Table 2). However, as only one examined individual demonstrated positive results for both organs (1/59), it is advisable that both organs should sampled to maximize the detection possibilities when there is no access to blood, which is the best material for this type of analysis [23].

The prevalence of *Anaplasma phagocytophilum* in wild boars varies across Europe and elsewhere, ranging from 0.97% in Belgium [24] to 44.8% in France [13]. In addition, one study reports that genetic material of *A. phagocytophilum* was not detected in the tested wild boar in Slovakia [25]. Although the prevalence of *A. phagocytophilum* identified in wild boars in the present study (20.34%) is consistent with the results of those carried out in Germany (12.5%) [26], Czech Republic and Japan (14.3%) [22, 27], Slovakia (28.2%) [2] and Hungary (39.2%) [1]; however, it is nevertheless one of the higher rates. By comparison, *A. phagocytophilum* was found to be present in 12% (39/325) of wild boars examined in west-central Poland (Mazovian Voivodeship) in 2012 [18].

Comparing the prevalence of *A. phagocytophilum* in wild boars in three regions of Poland, it can be seen that it is much more widespread in the west-central (12%) [18] and north-central (20.34%) regions than in the central (no positive samples) region (this study). The higher prevalence in the west-central and north-central regions may be due to the high afforestation density [28], which favors an increased incidence of ticks, these being known vectors of *A. phagocytophilum* [29]. The presence of increased numbers of vectors in an environment enables a faster spread of *A. phagocytophilum* among hosts. A similar correlation between geographic distribution and an increase in host prevalence has been shown by Szewczyk et al. [12].

Our sequencing of selected positive 16S rRNA partial gene samples (*n* = 4) confirmed the presence of *A. phagocytophilum* in the tested wild boars (MT510541). The identified nucleotide sequences are 100% identical to each other and to the 16S rRNA gene sequence of *A. phagocytophilum* isolates from various wild animals including carnivores (*Vulpes vulpes* MH328211, *Meles meles* MH328207, *Nyctereutes procyonoides* MH328209), wild boar (KM215225), cervids (*Capreolus capreolus* MN170723, *Cervus elaphus* KM215243) and small rodents (*Apodemus agrarius* KR611718, *Myodes glareolus* KC583437), as well as tick and flies (*Ixodes ricinus* HQ629922, JX173651, *Haematopota pluvialis* MH844585, *Tabanus distinguendus* MH844584), farm animals (*Bos taurus taurus* KP745629, *Equus caballus* AY527212), domestic animals (*Canis lupus familiaris* MN453474, MK814406) and humans (Belgium KM259921, Austria KT454992, USA AF093788, South Korea KP306518).

The two most common markers used to describe the genetic diversity of *A. phagocytophilum* strains are the *groEL* and *ankA* genes [10]. Based on our analysis of the *groEL* gene sequences (MT731760, MT731761, MT731762) the detected strains of *A. phagocytophilum* were classified into ecotype I [10, 30]. In the *groEL* gene, one nucleotide sequence (MT731760) showed 100% similarity to a *groEL* sequence from the human strain of *A. phagocytophilum* (AF033101), while another two (MT731761, MT731762) showed only 99.78%. However, despite the point change observed in the latter two sequences, all three encode the same protein as in human strains of *A. phagocytophilum*. Regarding *ankA*, two sequences (MT534241, MT731758), obtained from two wild boar individuals, are 100% complementary to human isolates (AF100886, AF100887, GU236800). The third isolate from wild boar (MT731759) showed 99.4% similarity to human markers. From all obtained *ankA* gene sequences, only two of them (MT534241 and MT731758) encode the same protein as human *A. phagocytophilum* strains: the third one (MT731759) is significantly different from the protein detected in humans. The analysis of the nucleotide sequences of 16S rRNA, *groEL* and *ankA* indicates that the strains of *A. phagocytophilum* detected in samples in this study can be classified as potentially zoonotic for humans.

## Conclusion

Our findings suggest that wild boars from Poland can be classified as a natural reservoir of the zoonotic *Anaplasma phagocytophilum* strains. In addition, both the spleen and liver were found to be suitable for the detection of *A. phagocytophilum*. However, further research is needed in other areas of Poland to comprehensively analyze the issues of *A. phagocytophilum* natural reservoirs throughout the country, and such studies should include other animals, which may demonstrate different tissue predilection than wild boars.

## Data Availability

The datasets generated during and/or analysed during the current study are available from the corresponding author on reasonable request.
